# Clinical Diagnosis in Veterinary Ophthalmology

**Published:** 1894-02

**Authors:** L. Van Es

**Affiliations:** Columbus, Nebraska


### CLINICAL DIAGNOSIS IN VETERINARY OPHTHALMOLOGY.

BY L. VAN ES, V.S.

Although the diseases of the eye and abnormalities of vision in the lower animals are by no means so frequent and important as in the human, we still should endeavor to be able to detect their exact nature.

Not only for proper treatment and prognosis, but also in forensic practice a proper diagnosis is of the highest value.

Before entering into details about the different diseases, a few remarks about the means and manner of examination will not be out of place.

As in all diagnostic examinations a certain systematic way should be followed, as eye and appendages are very complicated organs, and without a regular routine it is easy to overlook conditions of more or less importance.

First of all we must examine the eye with the naked eye and select a proper place to do so. Large animals we generally take in the doorway of a dark stall, the animal facing the light, while small animals may be placed upon a table, the patient also facing the light. Without touching the eye, we should see as to the conditions of the eyelids, injuries, tumors, the nature of the secretions (tears, mucus, pus, or a mixture of the three).

Having done this, proper attention is given to the size and shape of the bulb, comparing one eye with the other. In this manner conditions like bulbular atrophy (moon-blindness) and hydrophthalmus can be recognized.

The cornea should be examined very carefully and looked upon from all directions, so that we can well define the exact nature and situation of cloudiness, new growths, etc. Also the shape of the cornea must be looked upon.

The anterior chamber has to be tested as to its shape, size and contents; the aqueous humor must be clear and transparent. Pus forms a yellowish sediment in the most dependent part, and fibrinous exudates look like gray flakes, floating around in the chamber or adherent to its limiting structures. In examining the chamber we also detect the presence of anterior synechia and their extent. When the iris is normal, it must show its natural color, surface and reaction on light. In judging the color we must, of course, not think that the non-pigmented iris of wall-eyed horses has anything to do with disease. By the presence of inflammatory exudates, the iris takes a color from yellowish to gray, and by atrophic changes the color may be bluish gray.

Tubercular deposits on the iris have been seen in bovine patients.

The size and form of the pupil is an important factor in diagnosis. In amaurosis we have a large pupil and in most diseases of the middle coat the pupil is very much contracted. Posterior synechia are manifested by an irregular form of the pupil. In inflammatory diseases of the iris the reaction on light disappears or becomes weakened and slow. In amaurosis the pupil takes its maximum size, and light does not change it; the same condition we get after the application of a mydriatic. Of course synechia also greatly interfere with reaction on account of mechanical reasons.

The reflex from the tapetum lucidum also has a great diagnostic value, as any change in the refracting media will give rise to a change in appearance. Opacities in the lense are generally visible, but may be mistaken by the presence of images of surrounding objects; any motion of the head, however, will enable us to distinguish them.

In luxation of the crystalline lense, the iris will show a wave-like motion, and in this condition the background of the eye is sometimes visible. After seeing all we can, without touching the eye, the conjunctiva is to be examined by carefully lifting up the eyelids, and color, secretions, swelling, etc., noted. In diagnosis of inflammatory diseases of other organs, this examination is not without value.

Having the lids retracted, we take the opportunity to look after the condition of the blood-vessels of the sclerotic, as their being injected or not is of great importance in diagnosing disease of the uveal tract.

When carefully introducing a clean finger into the conjunctival sac, we are able to detect the presence of foreign bodies.

By palpitation of the eyeball we ascertain heat and the condition of the ocular tension.

In order to make an examination more minute, we can concentrate the rays of light on the parts with a convex lense or a concave mirror; this is called examination by focal illumination.

This is usually done in a dark place with artificial light, and by changing the position of the lense or mirror, light is concentrated on almost any part. For examination of the internal eye the ophthalmoscope is almost indispensable, although its use in our practice is as yet not so extensive as in human ophthalmology, where the exact nature of faulty refraction is diagnosed with the instrument. This, of course, can also be done in the animal eye, but our services are hardly ever required for these defects.

I will not enter into description of the instrument and the different kinds. As we all know, there are two methods of ophthalmoscopic examination, the direct and indirect. The first is done with a plain mirror and the latter with a concave one and a convex lense. The use of the plain mirror is to be recommended for veterinary practice, as in using the concave one we have to hold the lense close to the patient's eye, and this often induces him to close it. The indirect way, however, gives the advantages of a larger view; reason why this method is so much used in human ophthalmology.

In making an ophthalmoscopic examination, we generally use artificial light in a dark place, but daylight also does very well. We place the source of our light so that it does not illuminate the eye, but is easily caught by our mirror.

In using daylight we place the animal with its hind part towards a window or doorway and take care that no light comes in in front. It must be remembered that when using daylight the color of what we see through the ophthalmoscope always is lighter than when using artificial light.

Before entering into details on the different diseases, let us try and describe the ophthalmoscopic picture of a normal eye.

The first thing which strikes us when looking into a horse eye is the beautiful appearance of the tapetum, which varies in color in different animals and may be light green, yellowish-green, blue-reddish or nuances between these colors. The greenish tint is the most frequent and in this splendid colored field we find very small, dark spots which are formed by the blood-vessels of the choroid.

When looking lower down into the eye we come to line of demarcation between the tapetum lucidum and the tapetum nigrum, which has a very dark or black color.

In the tapetum nigrum, close to its edge, we notice the entrance of the optic nerve called papilla optici. It is well defined and oval in shape, the color is variable in different horses, but is chiefly composed of white, yellow or red. Blood-vessels sometimes give rise to small red spots near the centre.

Along the periphery of the papilla we notice the distributions of the *arteria centralis retinae*, which are very small and can only be traced for a small distance, especially at the lower edge of the papilla, where they are soon lost in the dark pigment.

There are many irregularities, which should not be mistaken for disease, as irregular edge of nerve head, spots, irregular line of demarcation between the tapetum lucidum and nigrum.

The best manner to avoid mistakes in that way is to examine large numbers of eyes and get familiar with the variations.

In dogs the tapetum and papilla are still more variable. The tapetum sometimes is beautiful gold-yellow, sometimes blue. The shape of the papilla varies from round to triangular and its color from light yellow to blue. The blood-vessels are large and can be followed all over the ophthalmoscopic field.

In the bovine the papilla is rather small, but the vessels are large and plainly visible. Prof. Möller suggests that in tuberculous cows the ophthalmoscopic examination may have some diagnostic value.

Sometimes it is advisable to dilate the pupil with atropia in order to get a larger view.

As diseases of the appendages of the eye very often have a certain degree of influence on the organ itself, we will mention a few of the most common pathological conditions of them.

Disease of the eyelids is uncommon, and if present a proper diagnosis will not be difficult to make. Wounds of the lids are generally of a lacerated nature, most frequent on the upper one. In the larger animals wounds are uncommon, but occasionally they are seen in dogs, often on account of being too familiar with their feline friends. In wounds of this nature we must see if the injury does not extend to the bulbus, which is often the case.

Blepharitis or inflammation of the eyelids is seen after blows or when the lid becomes involved in certain skin diseases. (Dogs).

It also can occur as an extension of a severe conjunctivitis. The trouble is recognized by the presence of increased heat and swelling, this sometimes to such an extent that the eye cannot be opened, and occasionally abscess formation is the result. The conditions known as ectropium and entropium is almost only seen in dogs, some breeds having a great tendency.

In ectropium the lid is turned outward, thus exposing the conjunctiva to the air with its impurities, which gives rise to a chronic conjunctivitis; it is mostly seen affecting the lower lid.

The opposite condition, known as entropium, most generally affects the upper lid. The lids will constantly be closed, profuse lachrymation, conjunctivitis, and by the irritation of the cornea, by the lashes sometimes, also keratitis.

The membrana nictitans often is the seat of disease. Conjunctivitis is liable to extend to this membrane, while injuries and new growths are not uncommon. Their diagnosis does not offer any difficulties. The condition known as "hooks" is a prolapse of the third eyelid.

Rarely we have to deal with disease or injury of the lachrymal apparatus, but as cases are on record we will mention the principal ones. When conjunctivitis extends over the mucous membrane, lining the lachrymal sac, it may give rise to a condition known as dacryocystitis. This is manifested by a fluctuating swelling over the region of the sac and by a muco-purulent discharge from the nasal canthus of the eye, caused by closure of the lachrymal canal. Pressing upward on the swelling, a discharge escapes from the puncta lachrymalia.

By breaking externally, dacryocystitis may give rise to a lachrymal fistula, which also may be caused by injury, but is very uncommon. The lachrymal canal can be obstructed by foreign bodies and by inflammatory processes, especially in connection with a chronic catarrh of nasal cavity and sinusses. There is a flow of tears, often mixed with pus, from the inner canthus. When we try to inject water through the canal we soon find out that the duct is obstructed.

Taking in consideration the structure and situation of the conjunctiva, it is not to be wondered at that conjunctivitis is one of the most frequent ocular diseases in the lower animals.

There are a number of distinct forms of conjunctivitis, which should be dealt with separately, as treatment is somewhat different. The simple inflammation (conjunctivitis catarrhalis) is generally caused by mechanical or chemical irritation, or is seen along with infectious diseases.

Redness of the conjunctiva and photophobia are about the first symptoms, and in more severe cases the lachrymation becomes profuse, the tears sometimes being mixed with pus and mucus. Frequently the inflammation extends over the cornea, which is recognized by a bluish color. It generally affects both eyes, except when due to a foreign body. In simple conjunctivitis the more superficial parts of the conjunctiva are affected.

When the deeper layers also become inflamed we speak of a conjunctivitis parenchymatosa. Along with the above mentioned symptoms we have a certain degree of swelling, on account of submucous exudates. These are often of a hæmorrhagic nature, due to severe injury, and give the parts an œdematous appearance. As a rule conjunctivitis is not accompanied by much pus formation, but in dogs and cattle a purulent form is sometimes seen in relation with corneal abscess. The lids are often found glued together, and there is a certain degree of itching, as the patients frequently rub and scratch the sore eyes. It must be looked upon as a serious condition, and along with keratitis it sometimes causes perforation of the cornea.

Under the name of conjunctivitis follicularis, Fröhner describes a form which, according to him, is very frequent in dogs. It is a chronic form and is characterized by a number of enlarged lymph-follicles, generally found on the inner side of the membrana nictitans, which often has the appearance of a granulating surface.

The diagnosis of disease or injury in the cornea is not very difficult. Wounds to the cornea are generally followed by photophobia and a profuse flow of tears. The extent of the injury should be carefully examined, by which the focal illumination has a great value. Around the wound we always notice a certain degree of cloudiness.

By perforation of the cornea, the aqueous humor will escape and the eye collapse. In some cases the iris will be forced against the corneal wound and act as a valve, a condition which may give rise to anterior synechia. If the wound is large the iris will sometimes prolapse. The perforation may be only partial and reach as far as the membrane of Descemet, which, with the aqueous humor behind it, often bulges through the external opening. In such a case we speak of a keratocele.

Keratitis or inflammation of the cornea is clinically to be divided in keratitis superficialis and keratitis profunda.

Keratitis superficialis generally follows conjunctivitis and is characterized by a diffuse opacity all over the corneal surface, which often has an uneven appearance on account of the extensive proliferation of epithelial cells. The color of the cornea varies from grayish blue to white; the whiter it is, the thicker the exudate. When the inflammation has lasted a certain length of time, small blood-vessels are seen penetrating the cornea along its margin.

In keratitis profunda, we have the inflammatory exudate within the corneal structure, in which it appears like gray or white spots. These will sometimes look like clouds, other times like streaks. Some writers describe a form in which we have several opaque spots with a lighter zone around it; this form is called keratitis punctata.

In very severe cases corneal abscess may be formed, which is recognized by its yellow color. This may result in a perforation, or when it breaks into the anterior chamber give rise to a condition known as hypopyon.

In both forms of keratitis we have, of course, the usual indications of irritation, as photophobia, lachrymation, etc.

Some four or five years ago, I witnessed in this State (Nebraska) an infectious eye disease among cattle, which has been designated as a keratitis infectiosa. I had occasion to watch it in a herd of dairy cows and in a number of calves.

The first symptoms were those of irritation, and in a few individuals lactation was considerably diminished. Among the little calves, I noticed some lose their usual appetite. Shortly after this the cornea became opaque and the conjunctivitis took a purulent nature. Abscess in the cornea was by no means uncommon, and several animals had their eyes destroyed by a purulent panophthalmitis, especially among the calves, of which a couple died in a cachexic condition. Some animals suffered only to a slight extent, and after a few days of impaired vision were all right again, but not a single one escaped the disease. Abscess in the cornea is sometimes followed by ulceration. In dogs, however, ulcerations are liable to follow distemper, and are seen in the intact cornea. It first appears like a small abrasion of the epithelial layer, but it very rapidly extends deeper and has the appearance of a small hole bored with a drill. The sides are straight down and the bottom is rough and gray colored. When the ulcer reaches the layer of Descemet, the fundus takes a bulging shape; there always is an extreme irritation. When the ulcer does not terminate in perforation it heals by granulation, and ultimately a cicatrix is formed, which often gives rise to a change in form of the eye. Pigmentary deposits in the corneal substance occur as a result of bleeding from the granulations. Under staphyloma we understand an abnormal projection of the cornea. The condition is not hard to be recognized, as the projection generally is very prominent, and sometimes conical in shape. Conditions like new growths or maculæ do not offer any difficulty in diagnosis, so we will not enter into details about them.

By its structure and anatomical situation the sclerotic coat is fairly well protected against external injuries and inflammatory processes. Wounds in that portion of the eye are very uncommon, and inflammation only seems to take place with panophthalmitis.

Of great importance for the veterinarian are the diseases of the middle coat of the eye, also called the uveal tract.

This coat, composed of the iris, ciliary bodies and choroid, is extremely vascular, and for this reason naturally predisposed to disease. Inflammation of the uveal tract can bring about very destructive changes in the tissue itself, but often also involves the lense, vitrous and retina. Inflammation of the iris (Iritis simplex) has an acute nature, as long as it does not involve the ciliary bodies and choroid, which sometimes is the case. There is a well marked injection of the sclerotic blood-vessels and rarely the cornea is clouded. The iris itself takes a gray color and fibrinous exudates are seen adherent to it or float around in the anterior chamber. The pupil is contracted, and reaction on light is slow and weak, while frequently there is formation of posterior synechia.

Along with conjunctivitis, iritis is seen in certain infectious diseases, and Möller says that in these cases there is a great tendency to hæmorrhagic extravasations.

The iris is sometimes involved in puncture of the cornea, in which case we speak of an iritis traumatica, a condition easy to be overlooked on account of the opaque cornea and extreme photophobia.

Closure of the pupil can be brought about by complete posterior synechia, and also by an over-development of the corpora nigra. Coloboma is the absence of a portion of the iris, and is the result of faults in the fœtal development, or of iridektomy.

Along with iritis, there may be present cyclitis or inflammation of the ciliary bodies, which can also take place without involving the iris. In such a case we have the injection of the sclerotic vessels, pain and photophobia, while there is a marked bulging of the iris in the ciliary region.

Chorioiditis is never seen apart from periodic ophthalmia, of which we now will study the symptoms.

Periodic ophthalmia is of the greatest importance to the veterinarian, as he is often called in to decide if a horse has moonblindness or not. The disease being of a recurrent nature, we have to be able to recognize the trouble during the acute attacks and in the intervals between them.

Let us see now what are the symptoms during the acute stage. There is a sudden manifestation of photophobia and profuse lachrymation. The seat of the disease principally being the uveal tract, the peri corneal injection is very well marked.

The conjunctiva is red, swollen, and along the periphery of the cornea there is a diffuse cloudiness, which gradually extends towards the centre till the transparency is almost entirely lost.

When the attack has lasted a certain time we notice small blood-vessels penetrating the cornea along its margin.

In cases where the transparency is not much interfered with we will find the iris rough on its surface and sometimes covered with an exudate. The pupil is much contracted and the reaction on light slow. The anterior chamber often is partly or entirely filled with an exudate, that appears to be pus, judging by its yellow color. The reflex from the tapetum is dark green, without any lustrum. Synechia are often present, and sometimes we are able to detect certain forms of cataract.

On palpitation we find the bulbus oculi generally very hard, as long as the disease has not caused bulbular atrophy.

With the aid of the ophthalmoscope, opacities in the vitrous are often seen in the earlier stages of the disease.

The acute attacks last from six to fourteen days, after which the eye may be all right to the casual observer. Often certain changes remain visible, like synechia, pigmentary deposits on the lense capsule, light color of the iris, green reflex from the pupil. Other changes can be seen with the ophthalmoscope, as opacities of the vitrous humor, cataract and detachments of the retina; when the pupil is dilated with atropia, it may be possible to detect them with the naked eye.

After the disease has made a certain amount of progress the bulb becomes atrophied, and is retracted in the orbit, thus giving rise to the wrinkled appearance of the upper lid, which is quite diagnostic. The recurrent nature of the attack is also a great help in diagnosis.

When examining between the attacks, the presence of cataract, opacities in the vitrous, or solution of the retina can, as a rule, be looked upon as resulting from periodic ophthalmia, and for this reason we will now describe their appearances.

When a cataract is complete it appears in the pupil as a gray or white spot, and offers no difficulty in detection. When it is less developed a more careful examination is necessary; the eye must be looked upon from different directions, and we have to watch that we do not mistake images of surrounding objects for opacities.

When examining by focal illumination the cataract is plainer seen, and when paying attention to the Purkinje-Sanson images, we can distinguish cataract from opacities in the vitrous.

The use of the ophthalmoscope is only indicated when the opacities are very small; they appear like black spots, caused by the fact that their being non-transparent hinders the light from being thrown on the retina.

From a clinical standpoint, it matters not what kind of cataract we have to deal with. Along with opacities in the vitrous we often find this humor liquified, which gives rise to the floating appearance of the opacities. When illuminating the eye properly we see them as gray spots, intercepting the reflex from the tapetum. When the opacity is diffuse it gives rise to an equal, dull green color.

To recognize detachments of the retina we generally have to use the ophthalmoscope. The retina has a gray or green appearance, and when the head is moved it often takes a wavy motion, while we sometimes can see folds in it. It is very hard sometimes to distinguish this condition from opacities in the vitrous. It is rarely that the trouble can be properly diagnosed, as opacities in the refracting media generally prevent us from doing so. Disease of the retina and optic nerve is not very liable to a successful treatment, but to make a reliable prognosis a correct diagnosis is indispensable.

In retinitis we have a contracted pupil, lachrymation, photophobia, while vision is much impaired.

When making an ophthalmoscopic examination we find the blood-vessels of the retina much engorged, and they seem to be more tortuous in their course. The retina is redder than usual, and when the disease has made some progress white spots or streaks can be seen.

Sometimes we also find the papilla involved in the inflammation, in which case we speak of neuroretinitis. The papilla is hyperæmic and swollen, having the same red color as the retina. When this condition lasts, it gives rise to atrophy of the papilla, owing to the interfered nutrition.

Opacities of the vitrous may be present, although to a lesser extent, as in inflammatory processes in the uveal tract.

Inflammatory disease in the orbit or in the cranial vault can be continued on the optic nerve (neuritis retrobulbularis). In such a case, we find vision very much interfered with, but there is not much photophobia or lachrymation, and the papilla is found to be engorged, while the retina generally is normal.

An atrophied papilla in the horse is white, very small, rounded as when normal the blood-vessels are very small and lesser in number.

When for any reason the opticus or its expansion, the retina, ceases to do its function we speak of amaurosis.

This is manifested by perfect blindness in the affected eye (sometimes on both). We find the pupil dilated to its maximum, while irritations, by means of strong light, have no influence. The reflex from the pupil is dark or dark blue.

Examination with the ophthalmoscope generally is without any results, and the great ophthalmologist, Beer, was very true in his statement, when he said that in amaurosis the doctor sees just as little as the patient.

Hæmorrhages in the retina are seen through the ophthalmoscope as dark red spots or streaks, and when severe this color is equally distributed all over the ophthalmoscopic field. Glaucoma is an ocular disease, which is only very rarely met with in veterinary practice. It has only been seen in dogs, and, as far as I can learn, it has never been observed in the horse.

There is an extreme hardness of the bulb, on account of increased intra-ocular pressure; the cornea may be slightly opaque, dim, and sometimes is in an anaesthetic condition. The iris is often found pressed against the cornea, while the pupil is dilated, the reflex being greenish in color.

The most diagnostic symptom however, is seen with the ophthalmoscope, in the form of a marked excavation of the optic papilla, caused by the great intra-ocular tension. The bottom of the excavation is seen by using a weak convex lense with the ophthalmoscope. As hydrophthalmus, a disease is known, manifested by an increased size of the bulb, due to an abnormal quantity of aqueous humor. The cornea generally is opaque, and becomes thin as a result of atrophic changes, due to pressure. There is danger of rupture and escape of the eye contents.

By panophthalmitis we understand a purulent inflammation, in which all the structures are involved. It generally is the result of punctured wounds or of pyæmia, resulting in embolism of the choroid vessels. The extreme inflammation along with profuse pus formation are always diagnostic.

There are certain parasites found in the eye; the irritation caused by their presence leads to a more minute examination, by which they are easily found.

Diseased conditions of the eye often coexist, which sometimes is rather confusing during examination, but generally there is no trouble in recognizing the exact condition. We always have to use a good deal of judgment in making our diagnosis.

COLUMBUS, NEBRASKA, December, 1893.

